# A Clinical Study of the Evaluation and Assessment of the Etiology and Patterns of Ocular Injuries in Midfacial Trauma in a Tertiary Care Hospital

**DOI:** 10.7759/cureus.10216

**Published:** 2020-09-02

**Authors:** Shrikar Umarane, Tejraj Kale, Arvind Tenagi, Yash Manavadaria, Abhishek S Motimath

**Affiliations:** 1 Oral and Maxillofacial Surgery, KLE Vishwanath Katti Institute of Dental Sciences, KLE Academy of Higher Education and Research, Belagavi, IND; 2 Ophthalmology, Jawaharlal Nehru Medical College, KLE Academy of Higher Education and Research, Belagavi, IND

**Keywords:** midfacial fractures, ocular injuries, maxillofacial injuries, eye signs

## Abstract

Aims

The aim is to study the pattern of ocular injuries in midfacial trauma and to evaluate the overall incidence of ophthalmic injury of any severity following maxillofacial trauma.

Methods and Materials

The maxillofacial surgeon conducted routine facial examination of patients with midfacial fractures, which also included a detailed ophthalmologic examination of patients, at the time of initial presentation. These patients were then further evaluated by an ophthalmologist for thorough examination of the eye.

Results

The total number of recorded midface maxillofacial trauma cases was 181. Out of 181 patients, 161 had ocular injuries. Among 181 cases, 161 (88.95%) cases were due to road traffic accidents, which was the prime etiologic factor. Out of total 181 patients, 172 (95.03%) were males and 9 (4.97%) were females. The maximum number of cases were of zygomaticomaxillary complex fractures (44.75%) followed by nasal bone fractures (21.5%). Periorbital ecchymosis accounted for the maximum number of cases, amounting to 61.88%. Loss of vision or blindness was seen in eight (4.42%) patients.

Conclusions

The study stresses further on the importance of long-term and continuous data collection and record management of trauma patients, which may help health care providers with necessary information to develop treatment protocols and device measures for the prevention of complications.

## Introduction

Maxillofacial fractures account for a substantial proportion in traumatology [1]. The severity of injuries ranges from a simple abrasion on the skin to tissue loss and complex facial fractures leading to cosmetic and functional discrepancy. Therefore, maxillofacial injuries may have a great amount of emotional, psychological, and financial impact on the patient because of the eventual disability and disfigurement.

The etiology of the trauma differs considerably according to the geographic location, culture, and socioeconomic status of the population. Road traffic accidents (RTAs) continue to be the leading cause, with assault coming close second. Recent studies indicated toward RTAs being the most frequent etiology in developing countries, whereas assault was the main cause in developed nations. Fall happens to be the most frequent cause of maxillofacial injuries in children and elderly individuals [1,2].

Several authors note that the most common site of maxillofacial fractures is the midfacial bones [3]. Maxillofacial trauma is often associated with serious concomitant injuries. Apart from the grave and life-threatening associated injuries, injury to the vital structures that the maxillofacial skeleton houses may lead to severe disability.

“Vision”, an important prime sense relies on the eye, is an organ occupying only 0.3% of total body surface [3]. In spite of the built-in protection (anatomy), ocular injuries causing significant functional and aesthetic defects are associated with 6-94% of maxillofacial injuries. Ocular injuries often accompany midface injuries. All facial trauma injuries, particularly above the level of the mouth, require a careful ophthalmic examination [4].

The degree of severity of ocular injuries to the eye and its adjacent structures varies a lot. The injuries range from contused lacerated wounds of the eyelids and corneal abrasions to wounds or rupture of the sclera, dislocation of the lens, intraorbital hemorrhages, and detachment of the retina. Globe rupture, optic nerve damage, derangement of the visual pathway as a result of retrobulbar hemorrhage, and perineural edema causing nerve compression subsequently leading to ischemic optic neuropathy remain to be the most commonly occurring ocular injuries. Hence, facial injuries particularly those affecting the midface warrant a careful examination of the eye by a specialist trauma team and eye surgeons.

The time of initial examination may be the only time when injuries to the vital structures, such as the retina and optic nerve, may be evident because later during the course of time, these signs become inconspicuous due to continuous hemorrhage. Some ocular injuries require immediate simultaneous surgical intervention of a maxillofacial surgeon and an ophthalmologist, whereas others may just render the immediate maxillofacial surgery contraindicated [4,5].

Last but not the least, recognition of an ocular injury before operation is important from a medico-legal point of view, as it ensures that the repair is not later attributed to be the cause of any permanent visual disturbance [4].

Few studies show a strong correlation between zygomaticomaxillary complex (ZMC) fractures and ocular injuries, whereas others point toward higher a incidence of ocular injuries with Le Fort III fractures. The prevalence of ocular injuries associated with facial fractures has been widely reported to be 2.7% to 90.6%. Overall, 95% of severe ocular injuries are associated with fractures of the facial middle third [6,7].

This descriptive study was designed to give a general idea of orbital fractures and ocular injuries associated with maxillofacial trauma. This will be a valuable aid in an early diagnosis and management of ocular injuries in midfacial trauma, which is important for the prevention of ocular dysfunction.

## Materials and methods

Study population

The study population consisted of 181 patients who reported to KLES Dr. Prabhakar Kore Hospital and Research Centre, Belagavi, Karnataka, India. The hospital is one of the tertiary referral centers for Belagavi district apart from other private and government institutes with a population of around 4.8 million according to the census of 2011.

Inclusion criteria

All patients with midfacial trauma, with or without other facial bone fractures who reported to KLES Dr. Prabhakar Kore Hospital and Research Centre in the period of September 2016 to May 2018 were included in the study after getting approval from the Ethical Committee of our institute.

Exclusion criteria

Patients reporting with pre-existing congenital or acquired ophthalmic disease or infections, ocular disorders such as cataract, glaucoma, and retinal disorders, and age-related macular degeneration (ARMD) were not a part of the study. This study did not include patients who received treatment in other health care facility apart from primary repair. Patients who were brought dead to the hospital or died during the course of treatment and those who were not willing for treatment and wanted to leave against medical advice were also excluded from the study.

Data collection

Simple random sampling technique was used for data collection. After a written informed consent from the patient was taken, maintaining the confidentiality, a pre-structured and pre-tested questionnaire was used to gather information followed by a thorough ocular examination. In cases where the condition of the victims did not warrant the interview, the relatives or attendants were interviewed. Medical records and case sheets were referred whenever necessary to collect additional information.

Statistical analysis

The data collected were analyzed using chi-square test and stratified according to age, sex, etiology, fracture type, and pattern of ocular injury.

Procedure

The maxillofacial surgeon conducted routine maxillofacial and oral examination of patients with clinically and radiographically proven midfacial fractures, which also included a detailed ophthalmologic examination of patients at the time of initial presentation. These patients were then further evaluated by an ophthalmologist for thorough examination of the eye.

## Results

The maxillofacial trauma was divided into ZMC fractures, Le Fort I, II, and III fractures, nasoorbitoethmoidal (NOE) fractures, orbital fractures, nasal fractures, zygomatic arch fractures, frontal bone fractures, and palatal fractures.

Etiology

Among 181 patients, RTAs were the primary cause of midfacial fractures (88.95%) (Table 1).

**Table 1:** Distribution of patients according to etiology of the midfacial fractures. RTA, road traffic accidents

Injuries	Number	Percentage
RTA	161	88.95
Assault	12	6.63
Fall	5	2.76
Others	3	1.66
Total	181	100.00

Within the category of RTAs, motorcycle accidents accounted for 115 (71.43%) cases followed by collisions with light and heavy vehicles that included 30 (18.63%) cases, and bicycle/bullock cart/pedestrian accidents that accounted for 16 (9.94%) cases (Table 2).

**Table 2:** Type of the RTA in patients with midface fracture. RTA, road traffic accidents

Type of RTA	Number	%
Motorcycle accident	115 (161)	71.43
Four-wheeler/bus/heavy vehicle	30 (161)	18.63
Bicycle/bullock cart/pedestrian hit	16 (161)	9.94

Upto 28.73% of patients were under the influence of alcohol at the time of injury, of which 52 were males.

Age distribution

Out of 181 patients, 66 (36.46%) were in the third decade of life and accounted for the maximum percentage of maxillofacial injuries followed by fourth (36 patients [19.89%]) and fifth decade (35 patients [19.34%]) of life. Patients below 20 years of age were less commonly affected, accounting only for 10.50% of the total. Only 4.42% of patients were from the age group of above 60 years (Table 3).

**Table 3:** Age-wise distribution of fractures.

Age groups	Number	Percentage
≤20 years	19	10.50
21-30 years	66	36.46
31-40 years	36	19.89
41-50 years	35	19.34
51-60 years	17	9.39
≥61 years	8	4.42
Total	181	100.00
Mean age	35.15	
SD age	13.61	

The right side was frequently affected, and ZMC fracture was the most commonly found fracture (48 patients [26.52%]) (Table 4).

**Table 4:** Fracture-wise distribution. R, right; L, left; B/L, bilateral; ZMC, zygomaticomaxillary complex; LF, Le Fort; NOE, nasoorbitoethmoidal

Fractures	Number	Percentage
R nasal	12	6.63
L nasal	6	3.31
B/L nasal	21	11.60
R ZMC	48	26.52
L ZMC	29	16.02
LF I	6	3.31
R LF I	0	0.00
L LF 1	1	0.55
LF II	10	5.52
R LF II	4	2.21
L LF II	3	1.66
LF III	7	3.87
R LF III	2	1.10
L LF III	0	0.00
R roof of the orbit	7	3.87
L roof of the orbit	11	6.08
R floor of the orbit	9	4.97
L floor of the orbit	3	1.66
R lateral wall of the orbit	6	3.31
L lateral wall of the orbit	5	2.76
R medial wall of the orbit	3	1.66
L medial wall of the orbit	1	0.55
R zygomatic arch	21	11.60
L zygomatic arch	15	8.29
Frontal bone	29	16.02
Palate	9	4.97
NOE	4	2.21

ZMC fractures were commonly seen in the third decade followed by the fourth and fifth decades. Most of the frontal bone fractures were also seen in the third decade of life (Table 5).

**Table 5:** Age range distribution of subjects as per the type of midfacial fracture. R, right; L, left; B/L, bilateral, ZMC, zygomaticomaxillary complex; LF, Le Fort; NOE, nasoorbitoethmoidal

Fractures	≤20 years	21-30 years	31-40 years	41-50 years	51-60 years	≥61 years	Total	%	Chi-square	p-Value
R nasal	4	2	3	1	2	0	12	6.63	10.0320	0.0740
L nasal	1	3	0	1	0	1	6	3.31	4.4830	0.4820
B/L nasal	3	9	3	3	1	2	21	11.60	3.2220	0.6660
R ZMC	3	17	12	8	6	2	48	26.52	2.9220	0.7120
L ZMC	4	13	3	7	2	0	29	16.02	4.7680	0.4450
LF I	0	2	0	3	0	1	6	3.31	7.6080	0.1790
R LF I	0	0	0	0	0	0	0	0.00	--	--
L LF 1	0	0	1	0	0	0	1	0.55	4.0500	0.5420
LF II	1	6	2	0	1	0	10	5.52	4.1290	0.5310
R LF II	0	2	1	1	0	0	4	2.21	1.3210	0.9330
L LF II	0	2	1	0	0	0	3	1.66	2.3720	0.7960
LF III	1	2	1	1	2	0	7	3.87	3.6090	0.6070
R LF III	1	0	1	0	0	0	2	1.10	5.3360	0.3760
L LF III	0	0	0	0	0	0	0	0.00	--	--
R roof of the orbit	0	2	1	2	1	1	7	3.87	3.1140	0.6820
L roof of the orbit	0	3	4	3	1	0	11	6.08	3.9990	0.5500
R floor of the orbit	0	4	1	2	0	2	9	4.97	9.2480	0.1000
L floor of the orbit	0	0	0	2	1	0	3	1.66	7.5700	0.1820
R lateral wall of the orbit	0	0	2	2	2	0	6	3.31	8.1680	0.1470
L lateral wall of the orbit	1	0	2	1	0	1	5	2.76	6.6710	0.2460
R medial wall of the orbit	1	0	0	1	1	0	3	1.66	5.5400	0.3540
L medial wall of the orbit	0	0	0	0	1	0	1	0.55	9.7010	0.0840
R zygomatic arch	2	4	7	3	5	0	21	11.60	10.7770	0.0560
L zygomatic arch	1	5	2	4	1	2	15	8.29	4.1500	0.5280
Frontal bone	2	16	3	3	4	1	29	16.02	7.5530	0.1830
Palate	2	4	1	1	1	0	9	4.97	2.5530	0.7690
NOE	0	3	0	0	1	0	4	2.21	4.9410	0.4230

Sex distribution

Out of total 181 patients, 172 (95.03%) were found to be males and 9 (4.97%) females. Most of the maxillofacial trauma cases were seen among males (Table 6).

**Table 6:** Gender-wise distribution of midfacial fractures.

Gender	Number	Percentage
Male	172	95.03
Female	9	4.97
Total	181	100.00

Maximum number of males sustained fractures in the third decade of life (64 patients), whereas females (3 patients) were more in the fifth decade of life (Table 7).

**Table 7:** Age-wise distribution of males and females who sustained midfacial fractures.

Age groups	Male	%	Female	%	Number
≤20 years	17	89.47	2	10.53	19
21-30 years	64	96.97	2	3.03	66
31-40 years	35	97.22	1	2.78	36
41-50 years	32	91.43	3	8.57	35
51-60 years	16	94.12	1	5.88	17
≥61 years	8	100.00	0	0.00	8
Total	172	95.03	9	4.97	181

Table 8 shows the distribution of males and females in different types of fractures. Maximum number of cases were of ZMC fractures (44.75%), accounting for a total of 73 cases in males and 4 cases in females. In the midface, nasal bone fracture cases were second highest in number, accounting for 39 patients.

**Table 8:** Association between genders with midfacial fractures. R, right; L, left; B/L, bilateral; ZMC, zygomaticomaxillary complex; LF, Le Fort; NOE, nasoorbitoethmoidal

Fractures	Male	Female	Total	%	Chi-square	p-Value
R nasal	12	0	12	6.63	0.6720	0.4120
L nasal	6	0	6	3.31	0.3250	0.5690
B/L nasal	21	0	21	11.60	1.2430	0.2650
R ZMC	47	1	48	26.52	1.1540	0.2830
L ZMC	26	3	29	16.02	2.1090	0.1460
LF I	6	0	6	3.31	0.3250	0.5690
R LF I	0	0	0	0.00	--	--
L LF 1	1	0	1	0.55	0.0530	0.8190
LF II	10	0	10	5.52	0.5540	0.4570
R LF II	3	1	4	2.21	3.4720	0.0620
L LF II	3	0	3	1.66	0.1600	0.6900
LF III	7	0	7	3.87	0.3810	0.5370
R LF III	2	0	2	1.10	0.1060	0.7450
L LF III	0	0	0	0.00	--	--
R roof of the orbit	7	0	7	3.87	0.3810	0.5370
L roof of the orbit	10	1	11	6.08	0.4200	0.5170
R floor of the orbit	9	0	9	4.97	0.4960	0.4810
L floor of the orbit	3	0	3	1.66	0.1600	0.6900
R lateral wall of the orbit	6	0	6	3.31	0.3250	0.5690
L lateral wall of the orbit	4	1	5	2.76	2.4580	0.1170
R medial wall of the orbit	3	0	3	1.66	0.1600	0.6900
L medial wall of the orbit	1	0	1	0.55	0.0530	0.8190
R zygomatic arch	20	1	21	11.60	0.0020	0.9620
L zygomatic arch	14	1	15	8.29	0.0990	0.7530
Frontal bone	29	0	29	16.02	1.8070	0.1790
Palate	9	0	9	4.97	0.4960	0.4810
NOE	4	0	4	2.21	0.2140	0.6440

Pattern of ocular injuries

**Figure 1:**
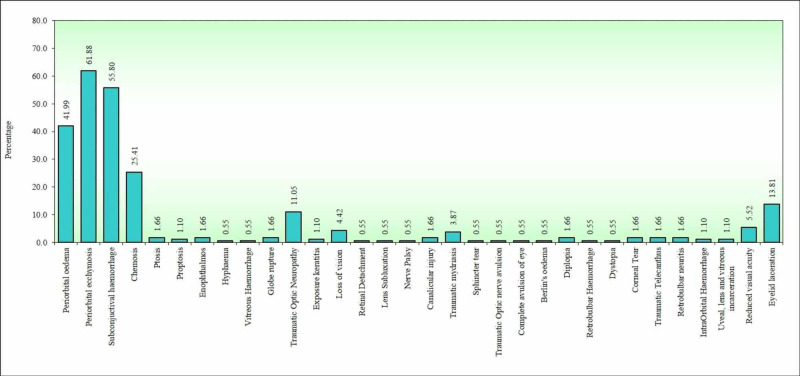
Ocular Injuries-wise distribution. *p<0.05

Figure 1 shows the number of patients associated with different types of ocular injuries. Periorbital ecchymosis accounted for the maximum number of cases, adding up to 61.88%, followed by periorbital edema and subconjunctival hemorrhage. Chemosis was seen in 25.41%, vitreous hemorrhage in 0.55%, traumatic optic neuropathy in 11.05%, corneal injury in 1.66%, reduced acuity in 5.52%, traumatic telecanthus in 1.66%, retrobulbar hemorrhage in 0.55%, diplopia in 1.66%, enophthalmos in 1.66% and hyphema in 0.55%. Globe rupture was seen in 1.66% and intraorbital hemorrhage in 1.10%. Retinal detachment was seen in 0.55%, lens subluxation in 0.55%, Berlin’s edema in 0.55%, and uveal, lens, and vitreous incarceration was seen in two (1.10%) cases. Proptosis and traumatic mydriasis occurred only in two (1.10%) patients and 1 (0.55%) patient, respectively. Traumatic III nerve palsy was seen in one patient and canalicular injuries were seen in three (1.66%) patients. Eyelid lacerations were seen in 25 (13.81%) cases. Loss of vision or blindness was seen in eight (4.42%) patients and was associated with different fracture patterns.

Most of the ocular injuries were seen in males (Table 9).

**Table 9:** Ocular injury and gender distribution.

Injuries	Male	Female	Chi-square	p-Value
Periorbital edema	70	6	2.3680	0.1240
Periorbital ecchymosis	106	6	0.0920	0.7620
Subconjunctival hemorrhage	97	4	0.4950	0.4820
Chemosis	45	1	1.0220	0.3120
Ptosis	3	0	0.1600	0.6900
Proptosis	2	0	0.1060	0.7450
Enophthalmos	3	0	0.1600	0.6900
Hyphema	1	0	0.0530	0.8190
Vitreous hemorrhage	1	0	0.0530	0.8190
Globe rupture	3	0	0.1600	0.6900
Traumatic optic neuropathy	20	0	1.1770	0.2780
Exposure keratitis	2	0	0.1060	0.7450
Loss of vision	7	1	1.0040	0.3160
Retinal detachment	1	0	0.0530	0.8190
Lens subluxation	1	0	0.0530	0.8190
Nerve palsy	1	0	0.0530	0.8190
Canalicular injury	3	0	0.1600	0.6900
Traumatic mydriasis	7	0	0.3810	0.5370
Sphincter tear	1	0	0.0530	0.8190
Traumatic optic nerve avulsion	1	0	0.0530	0.8190
Complete avulsion of eye	1	0	0.0530	0.8190
Berlin's edema	1	0	0.0530	0.8190
Diplopia	3	0	0.1600	0.6900
Retrobulbar hemorrhage	1	0	0.0530	0.8190
Dystopia	1	0	0.0530	0.8190
Corneal tear	3	0	0.1600	0.6900
Traumatic telecanthus	3	0	0.1600	0.6900
Retrobulbar neuritis	3	0	0.1600	0.6900
Intraorbital hemorrhage	2	0	0.1060	0.7450
Uveal, lens, and vitreous incarceration	2	0	0.1060	0.7450
Reduced visual acuity	10	0	0.5540	0.4570
Eyelid laceration	25	0	1.5180	0.2180

Maximum number of patients with ocular injuries were in the third decade of life followed by fifth and fourth decades. Ocular injuries were less commonly seen in patients in the sixth decade and in the age group of 20 years and below. Least number of ocular injuries were seen in the age group above 60 years (Table 10).

**Table 10:** Association between age groups and ocular injuries. *p<0.05.

Injuries	≤20 years	21-30 years	31-40 years	41-60 years	51-60 years	≥61 years	Chi-square	p-Value
Periorbital edema	6	28	15	16	9	2	2.8360	0.7250
Periorbital ecchymosis	13	44	19	21	12	3	4.8650	0.4330
Subconjunctival hemorrhage	12	37	18	21	10	3	2.3100	0.8050
Chemosis	5	18	4	10	5	4	6.8920	0.2290
Ptosis	0	3	0	0	0	0	5.3150	0.3790
Proptosis	0	0	0	0	0	2	43.7330	0.0001*
Enophthalmos	1	2	0	0	0	0	3.8970	0.5640
Hyphema	0	1	0	0	0	0	1.7520	0.8820
Vitreous hemorrhage	0	0	0	0	0	0	-	-
Globe rupture	0	0	3	0	0	0	12.2870	0.0310*
Traumatic optic neuropathy	2	9	2	2	1	4	15.3840	0.0090*
Exposure keratitis	0	2	0	0	0	0	3.5240	0.6200
Loss of vision	0	4	1	1	1	1	3.0540	0.6920
Retinal detachment	0	0	0	0	0	0	-	-
Lens subluxation	0	0	0	0	0	0	-	-
Nerve palsy	0	1	0	0	0	0	1.7520	0.8820
Canalicular injury	2	0	0	0	0	1	17.5340	0.0040*
Traumatic mydriasis	0	2	1	1	1	2	10.8950	0.0540
Sphincter tear	0	0	0	0	0	0	-	-
Traumatic optic nerve avulsion	0	0	1	0	0	0	4.0500	0.5420
Complete avulsion of eye	0	0	1	0	0	0	4.0500	0.5420
Berlin's edema	0	0	0	0	1	0	9.7010	0.0840
Diplopia	0	2	0	0	0	1	8.3360	0.1390
Retrobulbar hemorrhage	0	0	0	0	0		-	-
Dystopia	0	0	0	1	0	0	4.1950	0.5220
Corneal tear	0	1	0	1	1	0	3.2410	0.6630
Traumatic telecanthus	0	3	0	0	0	0	5.3150	0.3790
Retrobulbar neuritis	1	0	2	0	0	0	6.9950	0.2210
Intraorbital hemorrhage	0	1	0	0	0	1	10.8030	0.0550
Uveal, lens, and vitreous incarceration	0	0	2	0	0	0	8.1460	0.1480
Reduced visual acuity	1	5	0	1	0	3	19.7810	0.0010*
Eyelid laceration	2	9	6	4	4	0	3.2180	0.6660

Tables 11-13 show the distribution of ocular injuries in different types of fractures.

**Table 11:** Association between fractures and ocular injuries. R, right; L, left; B/L, bilateral; ZMC, zygomaticomaxillary complex; LF, Le Fort

Fractures	R nasal	L nasal	B/L nasal	R ZMC	L ZMC	LF I	R LF I	L LF I	LF II	R LF II	L LF II	LF III	R LF III	L LF III
Periorbital edema	5	2	5	23	14	2	0	1	8	3	3	2	1	0
Periorbital ecchymosis	7	3	11	31	25	2	0	1	8	3	3	6	2	0
Subconjunctival hemorrhage	4	3	5	31	22	3	0	1	8	3	2	6	2	0
Chemosis	5	2	3	12	7	3	0	0	2	1	1	2	0	0
Ptosis	0	0	0	0	0	0	0	0	0	0	0	0	0	0
Proptosis	0	0	0	0	0	0	0	0	0	0	0	0	0	0
Enophthalmos	0	0	0	0	1	0	0	0	0	0	0	1	0	0
Hyphema	0	0	0	0	0	0	0	0	0	0	0	1	0	0
Vitreous hemorrhage	0	0	0	0	0	0	0	0	0	0	0	1	0	0
Globe rupture	2	0	0	1	0	0	0	0	0	0	0	0	0	0
Traumatic optic neuropathy	1	2	3	5	2	0	0	0	1	1	1	3	0	0
Exposure keratitis	0	0	0	1	0	0	0	0	0	0	0	1	0	0
Loss of vision	1	0	1	3	0	0	0	1	0	0	0	0	1	0
Retinal detachment	0	0	0	0	0	0	0	0	0	0	0	0	1	0
Lens subluxation	0	0	0	0	0	0	0	0	0	0	0	0	1	0
Nerve palsy	0	0	0	1	0	0	0	0	0	0	0	0	0	0
Canalicular injury	1	0	1	0	0	0	0	0	0	0	0	0	0	0
Traumatic mydriasis	1	1	0	3	1	0	0	0	0	0	0	1	0	0
Sphincter tear	0	0	0	0	0	0	0	0	0	0	0	0	1	0
Traumatic optic nerve avulsion	0	0	0	1	0	0	0	0	0	0	0	0	0	0
Complete avulsion of eye	0	0	0	1	0	0	0	0	0	0	0	0	0	0
Berlin's edema	0	0	0	0	0	0	0	0	1	0	0	0	0	0
Diplopia	0	0	0	1	0	0	0	0	0	0	0	0	0	0
Retrobulbar hemorrhage	0	0	0	0	0	0	0	0	0	0	0	0	0	0
Dystopia	0	0	0	0	0	0	0	0	0	0	0	0	0	0
Corneal tear	0	0	0	0	0	0	0	0	1	0	0	0	0	0
Traumatic telecanthus	0	1	0	1	0	0	0	0	0	0	0	0	0	0
Retrobulbar neuritis	2	0	0	1	0	0	0	0	0	0	0	0	0	0
Intraorbital hemorrhage	0	0	0	0	0	0	0	0	0	0	0	1	0	0
Uveal, lens, and vitreous incarceration	2	0	0	0	0	0	0	0	0	0	0	0	0	0
Reduced visual acuity	1	1	1	0	1	0	0	0	0	0	0	1	0	0
Eyelid laceration	5	1	4	4	0	0	0	0	1	0	0	2	0	0

**Table 12:** Association between fractures and ocular injuries. R, right; L, left

Fractures	R roof of the orbit	L roof of the orbit	R floor of the orbit	L floor of the orbit	R lateral wall of the orbit	L lateral wall of the orbit	R medial wall of the orbit	L medial wall of the orbit
Periorbital edema	3	4	2	1	3	1	1	0
Periorbital ecchymosis	4	6	3	1	2	3	0	0
Subconjunctival hemorrhage	2	7	4	0	5	3	2	0
Chemosis	3	4	3	1	3	2	1	0
Ptosis	1	0	1	0	0	0	0	0
Proptosis	0	0	2	0	0	0	0	0
Enophthalmos	1	0	1	0	0	0	0	0
Hyphema	0	0	0	0	0	0	0	0
Vitreous hemorrhage	0	0	0	0	0	0	0	0
Globe rupture	0	0	0	0	0	0	0	0
Traumatic optic neuropathy	0	2	2	1	0	1	0	0
Exposure keratitis	0	0	0	0	0	0	0	0
Loss of vision	0	1	1	0	0	0	0	0
Retinal detachment	0	0	0	0	0	0	0	0
Lens subluxation	0	0	0	0	0	0	0	0
Nerve palsy	0	0	0	0	0	0	0	0
Canalicular injury	0	0	1	0	0	0	0	0
Traumatic mydriasis	2	0	1	1	1	0	0	0
Sphincter tear	0	0	0	0	0	0	0	0
Traumatic optic nerve avulsion	0	0	0	0	0	0	0	0
Complete avulsion of eye	0	0	0	0	0	0	0	0
Berlin's edema	0	0	0	0	0	0	0	0
Diplopia	1	0	1	0	0	0	0	0
Retrobulbar hemorrhage	0	0	0	0	0	0	0	0
Dystopia	0	0	1	0	0	0	0	0
Corneal tear	0	0	1	0	0	0	0	0
Traumatic telecanthus	0	0	0	0	0	0	0	0
Retrobulbar neuritis	0	0	0	0	0	0	0	0
Intraorbital hemorrhage	0	0	1	0	0	0	0	0
Uveal, lens, and vitreous incarceration	0	0	0	0	0	0	0	0
Reduced visual acuity	1	0	2	1	0	0	0	0
Eyelid laceration	1	2	1	1	1	1	0	0

**Table 13:** Association between fractures and ocular injuries. R, right; L, left; NOE, nasoorbitoethmoidal

Fractures	R zygomatic arch	L zygomatic arch	Frontal bone	Palate	NOE
Periorbital edema	8	4	10	4	1
Periorbital ecchymosis	9	8	19	6	2
Subconjunctival hemorrhage	4	5	16	6	1
Chemosis	3	4	7	3	1
Ptosis	1	0	1	0	1
Proptosis	0	0	0	0	0
Enophthalmos	0	0	1	0	0
Hyphema	0	0	0	0	0
Vitreous hemorrhage	0	0	0	0	0
Globe rupture	0	0	2	0	0
Traumatic optic neuropathy	0	4	4	1	1
Exposure keratitis	0	0	0	0	0
Loss of vision	0	2	1	0	0
Retinal detachment	0	0	0	0	0
Lens subluxation	0	0	0	0	0
Nerve palsy	0	0	0	0	0
Canalicular injury	0	0	0	0	0
Traumatic mydriasis	2	1	0	1	0
Sphincter tear	0	0	0	0	0
Traumatic optic nerve avulsion	0	0	0	0	0
Complete avulsion of eye	0	0	0	0	0
Berlin's edema	0	0	0	0	0
Diplopia	0	0	1	0	0
Retrobulbar hemorrhage	0	0	0	0	0
Dystopia	0	0	0	0	0
Corneal tear	0	0	1	0	1
Traumatic telecanthus	0	0	2	0	2
Retrobulbar neuritis	0	0	2	0	0
Intraorbital hemorrhage	0	0	1	0	0
Uveal, lens, and vitreous incarceration	0	0	2	0	0
Reduced visual acuity	0	1	3	0	1
Eyelid laceration	2	2	11	2	3

Maximum number of ocular injuries were seen in right ZMC fractures followed by left ZMC fractures and frontal bone fractures (Tables 11-13).

In the ZMC fracture cases treated for open reduction and internal fixation, there was no statistically significant difference between the heart rate pre-operatively and intra-operatively (Table 14).

**Table 14:** Heart rate pre-operative, intra-operative, and post-operative of ZMC fractures. ZMC, zygomaticomaxillary complex

Time points	Mean	SD	Mean difference	SD difference	Paired t-test	p-Value
Pre-operative	71.80	3.57				
At the time of reduction of fracture	72.16	3.20	-0.36	3.98	-0.7777	0.4392
Pre-operative	71.80	3.57				
Post-operative	72.26	3.19	-0.46	3.94	-1.0178	0.3120
At the time of reduction of fracture	72.16	3.20				
Post-operative	72.26	3.19	-0.11	4.67	-0.1964	0.8448

## Discussion

Orbital injury may lead to few of the most important complications following a maxillofacial trauma as it increases the risk of ocular trauma and optic nerve injury. The utmost importance of early diagnosis and intervention has been greatly highlighted by several studies particularly when there are definitive signs and symptoms of retrobulbar hemorrhage and increased intraocular tension. Even though the eye is fairly well protected against trauma by several factors such as eyelids, orbital rim, reflex actions such as blinking and hand over eye or head rotation away from the impact, facial fractures especially orbital fractures may still subject the patient at a risk of ocular injury [5,6].

The midfacial skeleton comprises the maxilla, frontal bone, zygoma, zygomatic arch, palatine bone, vomer, and nasal bones. Sphenoid, frontal, and ethmoid bones may not be part of facial structures anatomically but are frequently involved in midfacial fractures. Midfacial fractures are commonly classified as Le Fort I, II, and III fractures, ZMC, zygomatic arch fractures, NOE fractures, nasal bone fractures, and fractures of the palate and frontal bone. These may occur in isolation or together [1,6].

Ocular injuries in association with facial trauma can be attributed to variable etiology. These can be due to sports, industrial hazards, or assault, and can also be self-inflicted. In all high-speed RTAs, the injuries usually are grave and lead to orbital fractures and eventually injury to the eyeball and associated structures. Having a sound knowledge of association of ocular injuries and its types and frequency plays a pivotal role in giving a wholesome treatment to the patient. If the diagnosis is missed, it will influence the overall treatment and ultimate prognosis of the patient [5,6,8].

The demographic data and pattern of maxillofacial fractures vary according to various socioeconomic and geographic factors [2,8]. When it comes to etiology, RTAs remain to be the leading cause of maxillofacial trauma [9,10]. Studies conducted in developed nations have suggested a changing trend in the etiology of maxillofacial trauma from RTA to assault, with fall being the most common etiology [9,10]. Septa et al. reported the incidence or maxillofacial trauma due to RTA to be 64%. In RTA, two-wheelers appear to be the major cause of accident, with accidents due to motorcycles accounting for 55.4% [8]. A total of 181 patients with midfacial fractures were included in our study. In our study, 115 cases (71.43%) of RTA due to two-wheelers were seen followed by four-wheelers and other heavy vehicles (18.63%) and 16 cases wherein the victim was a pedestrian or in a bullock cart (16 patients [9.94%]). Septa et al. reported that 33.5% of patients were under alcohol influence, with males accounting for 99% [8]. In our study, 28.73% were under the influence of alcohol and all of them were males (52 patients). Thus, there should be strong pressure by governments through advertisement and television to outlaw drunk drivers and enforce regulation.

Majority of the maxillofacial trauma cases were seen in patients in the third decade of life followed by fourth and fifth decades. First and seventh decades of life accounted for least number of cases. These findings are supported by various studies in literature suggesting maxillofacial injuries to be more common in the second and third decades of life [11,12]. Low incidence of maxillofacial injuries have also been reported by some authors in age groups below 10 years and above 60 years [13,14].

Of the total patients recorded, 95.03% were male and 4.97% were females. The ratio of males to females was 19.11:1. These values in other studies show that the ratio ranged from 2.19:1 to as high as 11.8:1. Males are shown to be more susceptible to maxillofacial trauma in almost all the previous studies [15,16].

Most common midfacial fracture recorded in our study was of ZMC (44.75%) followed by nasal fractures (21.5%). Le I, II, and III fractures accounted for 3.86%, 9.39%, and 3.86%, respectively. Frontal bone fractures accounted for 16.02%. NOE fractures accounted for 2.21%. ZMC fractures comprising the maximum number of cases is a finding that is concurrent with other studies [1,16,17]. The incidence of Le Fort fractures reported by us was quite low compared to studies conducted by other authors [18-20].

Occurrence of concomitant ocular injuries with maxillofacial fractures may range from as low as 2.7% to as high as 90% [21-24]. In our study, out of the total 181 patients, 161 (88.9%) patients had some type of ocular injuries. It falls within the range documented by other authors as mentioned above. Such a wide range of reported incidence of concomitant ocular injuries maybe due to difference in types of ocular injuries included in the study and difference in expertise of the examiner and specificity of the examination performed.

Periorbital ecchymosis (61.88%) accounted for the maximum number of total ocular injuries followed by periorbital edema (41.99%) and subconjunctival hemorrhage (55.80%). Chemosis was seen in 25.41%, vitreous hemorrhage in 0.55%, traumatic optic neuropathy in 11.05%, corneal injury in 1.66%, reduced acuity in 5.52%, traumatic telecanthus in 1.66%, retrobulbar hemorrhage in 0.55%, diplopia in 1.66%, enophthalmos in 1.66% and hyphema in 0.55%. Globe rupture was seen in 1.66% and intraorbital hemorrhage in 1.10%. Retinal detachment was seen in 0.55%, lens subluxation in 0.55%, Berlin’s edema in 0.55%, and uveal, lens, and vitreous incarceration in 1.10%. Proptosis and traumatic mydriasis occurred only in two (1.10%) patients and one (0.55%) patient, respectively. Traumatic III nerve palsy was seen in one patient, canalicular injuries in three (1.66%) patients, and eyelid lacerations in 25 (13.81%) patients. Loss of vision or blindness was seen in eight (4.42%) patients.

Subconjunctival hemorrhage being one of the most common ocular injuries in our study compares favorably with other studies, where it is found to be between 60 and 74% [25,26]. Of the total 181 cases of subconjunctival hemorrhage, 101 (55.80%) were found in association with ZMC fractures, Le Fort fractures, frontal bone fractures, nasal bone fractures, and orbital fractures.

Diplopia is one of the common findings in patients of maxillofacial fractures. The incidence of diplopia was found to be 1.66% in our study, which is significantly less than the incidence reported by other studies, which was around 19% [25,27,28]. Septa et al. reported 11.5% patients with diplopia [8]. Mittal et al. reported a 60% incidence of diplopia in orbital fractures, and rest 40% were seen in ZMC fractures [5]. In our study, ZMC fractures, orbital floor fractures, and orbital roof fractures were associated with diplopia cases.

Enophthalmos following a maxillofacial trauma is a definitive indication of radiological imaging to confirm fractures of orbital wall. CT scan being more specific is advised over plain X-ray. In our study, a total of 1.66% patients presented with enophthalmos, which is on the lower side when compared to figures (8%) reported by Al-Qurainy et al. [28]. Septa et al. reported an incidence of 8.5% for enophthalmos [8]. Amrith et al. recorded a 6% incidence of enophthalmos, with 81% of it occurring in orbital fracture patients [22]. In our study, out of three cases of enophthalmos, one was associated with orbital floor and roof fractures, one was associated with ZMC fractures, and one was associated with Le Fort III fractures.

All patients with maxillofacial injuries should mandatorily be checked for pupillary reflex and visual acuity. These are often called as “vital signs” of the eye. We found reduced visual acuity to be present in 5.52% of patients at the time of injury, which is near to the 11.5% given by Septa et al. [8]. Al-Qurainy et al. found visual acuity to be reduced in around 15% of the patients [28]. Amrith et al. reported 23% patients with reduced visual acuity, with 12.5% having permanent visual impairment [22]. In our study, 10 out of 181 patients of reduced visual acuity were having nasal bone fractures, Le Fort III fractures, frontal bone fractures, floor of the orbit fractures, and ZMC fractures. These findings suggest that reduction in visual capacity is usually associated more with higher level of complex midfacial fractures.

Total loss of vision or blindness is an uncommon sequela of maxillofacial fractures. A total of eight cases of blindness were recorded by us out of 181 patients, with the incidence being 4.42%. One case was due to complete avulsion of the globe, two were due to globe rupture, two due to intraorbital hemorrhage, one due to retrobulbar neuritis, one due to traumatic optic neuropathy, one due to lens subluxation and retinal detachment, and one due to uveal, lens, and vitreous incarceration. The incidence reported in literature is between 0.3% and 3.5% [8,21,23,24]. The loss of vision could be consequential to injury to the globe, optic nerve, or visual pathway.

Tearing of blood vessels at the root of the iris may lead to hyphema or blood in the anterior chamber of the eye. We found the incidence of hyphema to be 0.55% and was seen in Le Fort III fractures. The incidence relates to the findings of other studies such as those done by Septa et al. and Al-Qurainy et al., and few others, who reported it to be around 3.5% [8,21,23,25].

Traumatic telecanthus is detachment of the medial canthus of the eye. In this study, 1.66% patients presented with traumatic telecanthus, whereas the findings of the study conducted by Septa et al. showed 5% [8]. In our study, the highest incidence of traumatic telecanthus was seen in NOE fractures.

Three (1.66%) patients were found to have corneal tear, two (1.10%) patients were found to have proptosis, and seven (3.87%) patients were found to have traumatic mydriasis. The low incidence of these injuries is in accordance with the findings by Septa et al. It suggests that retinal tear, proptosis, and traumatic mydriasis are not common ocular injuries associated maxillofacial trauma [8]. Eyelid lacerations were present in 25 (13.81%) cases, for which meticulous suturing was planned.

Al-Qurainy et al. classified the ocular injuries into mild, moderate, and severe ophthalmic injuries [21]. Among these, our study had minor ophthalmic disorders such as eyelid swelling and bruising, subconjunctival hemorrhage, chemosis, and commotio retinae (Berlin’s edema). Moderate ophthalmic injuries such as enophthalmos, eyelid lacerations, traumatic pupillary changes, and lens damage were seen. Severe ophthalmic disorders included retrobulbar hemorrhage, hyphema, optic nerve injury, vitreous hemorrhage, and retinal detachment. Jamal et al. reported that 66.6% had minor ocular injuries and 10% had major ocular injuries such as ruptured globe and retinal hemorrhage [26].

In our study, ZMC fractures were associated with periorbital edema, periorbital ecchymosis, subconjunctival hemorrhage, and chemosis. Enophthalmos was seen in one case. Globe rupture and complete avulsion of the eye along with traumatic optic nerve avulsion and retrobulbar neuritis were seen in three individual cases, which led to loss of vision in these cases. All three fractures were seen in males. Exposure keratitis was also seen in one ZMC case. In the cases taken up for open reduction and internal fixation of ZMC fractures, there was no statistically significant difference between the heart rate pre-operatively and at the time of reduction of the fracture, indicating that was no significant change in the heart rate (bradycardia secondary to oculocardiac reflex during reduction of ZMC fractures) which is due to the anesthetic agents and the local infiltration of adrenaline that is given [29].

Nasal bone fractures (adding up left, right, and bilateral nasal bone fractures as shown in Table 4) accounted for the second most common midfacial fracture in our study, wherein two cases of globe rupture were seen along with other fractures leading to blindness. Canalicular injury and traumatic mydriasis were seen in two cases each, and traumatic optic neuropathy was noted in six cases. Two cases of uveal, lens, and vitreous incarceration were also seen along with nasal bone fractures.

The incidence of ocular injuries in Le Fort I fractures was very less in this study.

In association with Le Fort II fractures, three cases had traumatic optic neuropathy, one case had Berlin’s edema, and one case had a corneal tear.

Le Fort III fractures were associated with a considerable number of ocular injuries ranging from subconjunctival hemorrhage, chemosis, traumatic optic neuropathy, traumatic mydriasis, and intraorbital hemorrhage.

NOE fractures presented clinically with traumatic telecanthus. Corneal tear was also diagnosed in one case of NOE fracture in our study. Frontal bone fractures also presented with a variety of ocular disorders such as diplopia, corneal tear, retrobulbar neuritis, and traumatic optic neuropathy.

Orbital fractures, especially the roof and the floor of the orbit fractures, presented with ocular injuries such as dystopia, loss of vision, intraorbital hemorrhage, and diplopia.

It is very essential to note that some of these fractures presented with some ocular injuries, which required timely management. It is necessary to not only know the common ocular injuries pertaining to a said fracture but also certain uncommon eye injuries as seen in our study.

It has been suggested that there is an increased risk of ocular injuries with maxillofacial trauma by a factor of 6.7 as compared to other major trauma patients with no facial injury [30]. The difference in the incidence of different types of ocular injuries in various studies is probably because some injuries may have gone undetected or may have been neglected in view of more significant ocular injuries also being present.

This study strives to draw attention toward concomitant ocular injuries in maxillofacial trauma patients, which may go unnoticed and later present as a serious complication incapacitating the patient. Long-term and continuous record management can make it possible to correlate ocular and orbital injures in maxillofacial trauma patients.

## Conclusions

This prospective study was conducted to have an idea about concomitant orbital and ocular injuries in patients who sustained maxillofacial trauma. It is clear from this study that there is a very high probability of associated ophthalmic injuries in patients with midfacial injuries. Majority of the patients had associated eye injuries. The study stresses further on the importance of long-term and continuous data collection and record management of trauma patients, which may help health care providers with necessary information for the development of treatment protocols and device measures for the prevention of complications. The general population can be educated regarding the importance of obeying traffic rules and following road safety instructions with the help of such data.
